# Usability of serum annexin A7 as a biochemical marker of poor outcome and early neurological deterioration after acute primary intracerebral hemorrhage: A prospective cohort study

**DOI:** 10.3389/fneur.2022.954631

**Published:** 2022-08-08

**Authors:** Chuan-Liu Wang, Yan-Wen Xu, Xin-Jiang Yan, Cheng-Liang Zhang

**Affiliations:** ^1^Department of Neurology, The Quzhou Affiliated Hospital of Wenzhou Medical University, Quzhou People's Hospital, Quzhou, China; ^2^Department of Neurosurgery, The Quzhou Affiliated Hospital of Wenzhou Medical University, Quzhou People's Hospital, Quzhou, China

**Keywords:** biomarkers, intracerebral hemorrhage, severity, functional outcome, early neurological deterioration, annexin A7

## Abstract

**Objective:**

Annexin A7 (ANXA7), a calcium-dependent phospholipid-binding protein, may act to aggravate brain injury. This study aimed to assess the clinical utility of serum ANXA7 as a predictor of severity, early neurological deterioration (END), and prognosis after intracerebral hemorrhage (ICH).

**Methods:**

A total of 126 ICH patients and 126 healthy controls were enrolled. Symptomatic severity was evaluated utilizing the National Institutes of Health Stroke Scale (NIHSS) score. The lesion volume of ICH was measured according to the ABC/2 method. END was referred to as an increase of 4 or greater points in the NIHSS score or death at post-stroke 24 h. The unfavorable functional outcome was a combination of death and major disability at post-stroke 90 days.

**Results:**

Serum ANXA7 levels were significantly higher in patients than in controls (median, 46.5 vs. 9.7 ng/ml; *P* < 0.001). Serum ANXA7 levels were independently correlated with NIHSS score [beta: 0.821; 95% confidence interval (CI): 0.106–1.514; variance inflation factor: 5.180; *t* = 2.573; *P* = 0.014] and hematoma volume (beta: 0.794; 95% CI: 0.418–1.173; variance inflation factor: 5.281; *t* = 2.781; *P* = 0.007). Serum ANXA7 levels were significantly elevated with increase in modified Rankin scale scores (*P* < 0.001). Also, serum ANXA7, which was identified as a categorical variable, independently predicted END and an unfavorable outcome with odds ratio values of 3.958 (95% CI: 1.290–12.143; *P* = 0.016) and 2.755 (95% CI: 1.051–7.220; *P* = 0.039), respectively. Moreover, serum ANXA7 levels efficiently differentiated END (area under the curve: 0.781; 95% CI: 0.698–0.849) and an unfavorable outcome (area under the curve: 0.776; 95% CI: 0.693–0.846).

**Conclusion:**

Serum ANXA7 may represent a useful blood-derived biomarker for assessing the severity, END, and prognosis of ICH.

## Introduction

Intracerebral hemorrhage (ICH), one of the most severe forms of stroke, accounts for 15–20% of all strokes and is characterized by high morbidity and mortality (1). After ICH, blood rapidly accumulates within the surrounding brain, thereby resulting in primary brain injury (2). Afterward, secondary brain injury is induced, which involves inflammatory response, neuronal apoptosis, disrupted blood-brain barrier, and brain edema, finally resulting in neurological dysfunction (2). In general, the National Institutes of Health Stroke Scale (NIHSS) score, Glasgow Coma Scale (GCS) score, ICH score, and bleeding size are the four most common prognostic determinants of ICH (3–5). Early neurological deterioration (END) is an adverse event often encountered during the early period of ICH, and its occurrence markedly increases the risk of poor outcome (6). In recent decades, a growing body of study has focused on exploring blood-derived biochemical markers to facilitate the development of END and outcome predictions after ICH (7, 8).

Annexins are well-known as calcium-dependent phospholipid-binding proteins and are implicated in multiple cellular functions, including membrane trafficking, Ca^2+^ homeostasis, and exocytosis (9). Annexin A7 (ANXA7), also named synexin, is the first isolated annexin (10). In the central nervous system, ANXA7 was found mainly in human and animal neurons (11–15). Moreover, its expression was significantly upregulated in brain tissues of rats with traumatic brain injury (11), ICH (12), subarachnoid hemorrhage (13), or acquired epilepsy (14), and even in those of refractory epilepsy patients (15). Experimental data showed that ANXA7 may function to aggravate brain damage *via* affecting glutamate release, inducing neuronal apoptosis, disrupting the blood–brain barrier, and increasing brain edema, thereby worsening neurological function after traumatic brain injury (11), ICH (12), and subarachnoid hemorrhage (13). Hence, it is postulated that ANXA7 may be a biomarker of acute brain injury. However, to the best of our knowledge, ANXA7 has not been measured in human peripheral blood after acute brain injury. This study was designed to assess whether serum ANXA7 levels are associated with illness severity, END, and neurological functional outcome after ICH.

## Materials and methods

### Study design and participant selection

In this prospective, cohort study performed at our hospital between May 2018 and May 2021, patients with non-traumatic first-ever ICH were consecutively enrolled. Next, we excluded those patients with age of <18 years, hospital admission of >24 h after stroke onset, a surgical evacuation of hematoma, primary intraventricular hemorrhage, bleedings resulting from other causes (e.g., intracranial tumor, cerebral arteriovenous malformation, hemorrhagic transformation of cerebral infarction, moyamoya disease, and intracranial aneurysm), history of neurological diseases (for instance, ischemic or hemorrhagic stroke, severe traumatic brain injury, and multiple sclerosis), or other specific diseases or conditions (such as autoimmune diseases, malignancies, pregnancies, uremia, liver cirrhosis, and chronic heart or lung disease). In addition, a group of healthy volunteers was recruited as controls.

This study was carried out in compliance with the tenets of the Declaration of Helsinki and the protocol confirmed with the ethical standards of our hospital on human subjects. The study protocol was approved by the Human Investigations Committee at our hospital. We acquired written informed consent to participate in this study from legal representatives of patients or controls themselves.

### Clinical assessment and biochemical investigations

Upon arrival at the emergency center, we collected some data including demographics (age and gender), vascular risk factors (hypertension, diabetes mellitus, and hyperlipidemia), lifestyle risk factors (cigarette smoking and alcohol drinking), medication history (use of statins, anticoagulation drugs, and antiplatelet drugs), anthropometric measurements (systolic blood pressure, diastolic blood pressure, and body mass index), and time from stroke onset to hospital admission. The stroke symptomatic severity was assessed using the NIHSS (16). The severity of the stroke consciousness was evaluated using GCS (17). Hematoma volume was estimated in accordance with the ABC/2 method (18). Hematoma locations were divided into infratentorial and lobar hemorrhages. Extension of hematoma into the intraventricular cavity or subarachnoidal space was also recorded. ICH score was estimated based on age, hematoma volume, GCS score, infratentorial hemorrhage, and extension of hematoma into the intraventricular cavity (3). END was referred to as an increase of ≥4 points in the NIHSS score or death at 24 h from symptoms onset (19). ICH patients were followed up until death or completion of 90 days after ICH. An unfavorable outcome was defined as a combination of death and major disability [modified Rankin Scale score (mRS) of 3 or greater] (20).

Venous blood samples were obtained from ICH patients at emergency center and from controls at entry into the study. We performed some routine blood tests, including white blood cell count, serum C-reactive protein levels, and blood glucose levels. In order to determine ANXA7 levels, serum was stored at −80°C until assayed. The same technician, who was blinded to study data, employed a commercial enzyme-linked immunosorbent assay kit (Shanghai Beinuo Biotechnology Co., Ltd, China) to gauge serum ANXA7 levels in all participants based on the manufacturer's instructions. Samples were measured in duplicate, and the mean value of two measurements was utilized for further statistical analysis.

### Statistical analysis

The statistical software used in this study were the Statistical Package for the Social Sciences version 19.0 (SPSS Inc., Chicago, IL, United States) and MedCalc 9.6.4.0 (MedCalc Software, Mariakerke, Belgium), and graphs were plotted using the GraphPad Prism 5.0 software (GraphPad Software, La Jolla, CA, United States). Qualitative variables were shown as counts (percentages). After the Kolmogorov–Smirnov test, normally and non-normally distributed quantitative data were presented as means ± standard deviations and medians (lower-upper quartiles), respectively. The differences in baseline demographic, clinical, radiological, and biochemical data between the two groups were determined using the chi-square test, Fisher's exact test, independent *t*-test, or Mann–Whitney *U* test where appropriate. Serum ANXA7 levels were compared among multiple groups using Kruskal–Wallis *H* test. Spearman correlation analysis was carried out to assess the bivariate correlations between serum ANXA7 levels and other variables, and *ρ* values were reported for showing correlations. The multivariate linear regression model, which included other significantly pertinent variables (*P* < 0.05) in univariate correlation analysis, was built to determine factors, which were independently correlated with serum ANXA7 levels. The influence of serum ANXA7 levels on the occurrence of END or the development of an unfavorable outcome was investigated using multivariate logistic regression analysis, which allowed adjustment for some conventional confounding factors (e.g., age, NIHSS scores, hematoma volume, GCS scores, and intraventricular hemorrhage), which were also significant (*P* < 0.05) on univariate analysis. The results were reported as odds ratio (OR) and 95% confidence interval (CI) to show associations. However, as the ICH score was composed of 5 subcomponents, namely, age, hematoma volume, GCS score, infratentorial hemorrhage, and extension of hematoma into the intraventricular cavity, the ICH score was not incorporated into the multivariate model. Area under the receiver operating characteristic (ROC) curve (AUC) and the corresponding 95% CI were calculated to reflect the predictive ability. Using the Youden method, an optimal value of serum ANXA7 levels was selected, which generated the corresponding sensitivity and specificity values. A *P*-value of <0.05 indicated significance.

## Results

### Participant characteristics

**Figure 1:**
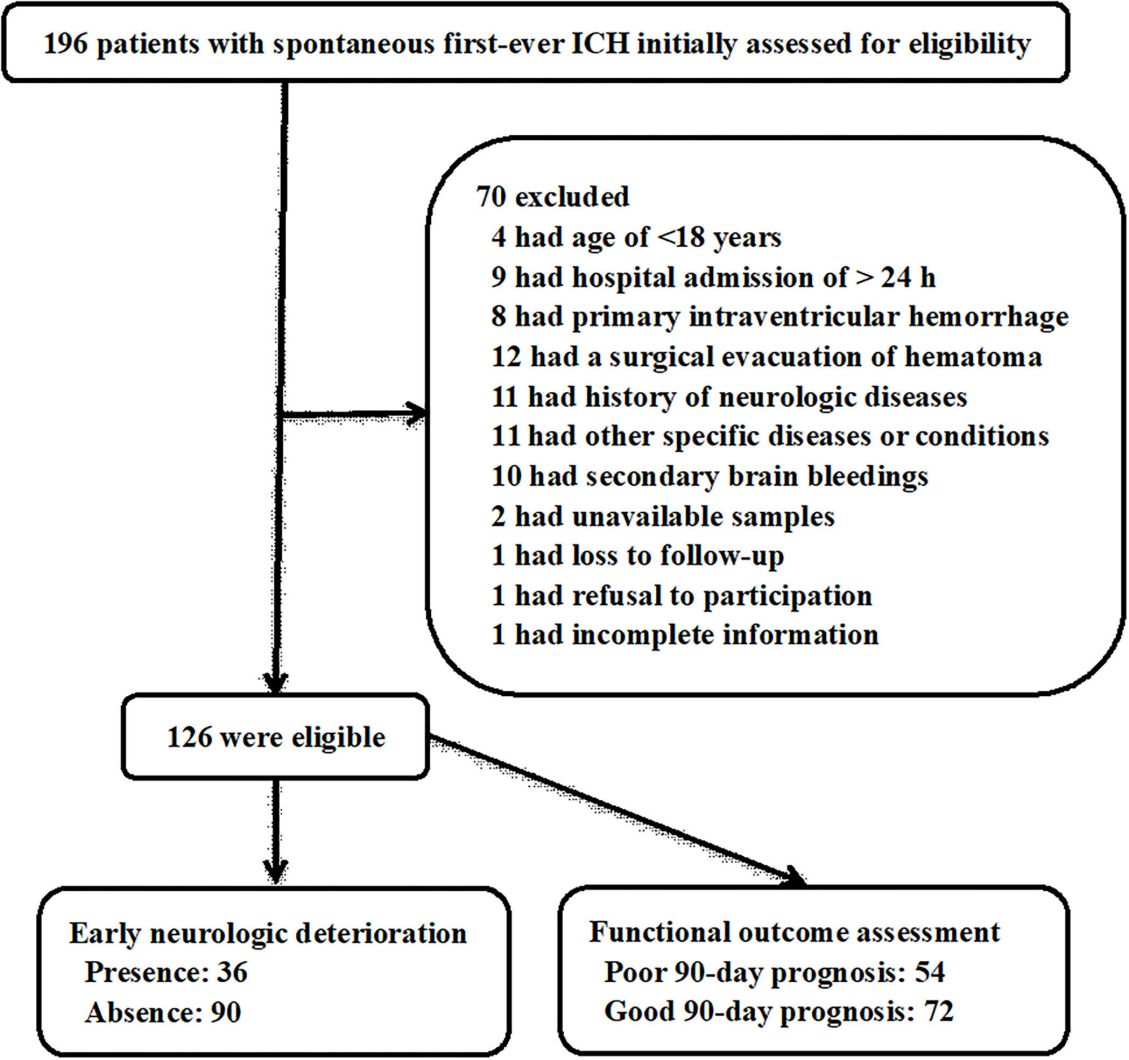
Flowchart for selecting eligible patients with spontaneous intracerebral hemorrhage. A total of 196 intracerebral hemorrhage patients were first screened and then 70 of them were excluded from this study. Finally, a total of 126 patients were eligible for further analysis, among whom, there were 36 patients with early neurological deterioration and 54 with poor prognosis at 90 days after stroke. ICH, intracerebral hemorrhage.

In this study, we first enrolled a total of 196 patients with non-traumatic first-ever ICH and thereafter excluded 70 patients due to reasons that are outlined in Figure 1. Ultimately, a total of 126 eligible ICH patients were included in the study analysis. In addition, 126 healthy controls were enrolled, who were aged from 24 to 88 years (mean, 58.1 years; standard deviation, 14.3 years) and included 68 males and 58 females. Age and gender did not differ significantly between 126 ICH patients and 126 healthy controls (both *P* > 0.05).

The stroke patients, 70 being males and 56 being females, were aged from 40 to 82 years (mean, 59.6 years; standard deviation, 10.9 years). Body mass index ranged from 19.7 to 30.3 kg/m^2^ (mean, 25.0 kg/m^2^; standard deviation, 1.9 kg/m^2^). Vascular risk factors included hypertension (78 patients, 61.9%), diabetes mellitus (20 patients, 15.9%), and hyperlipidemia (36 patients, 28.6%). Adverse lift habits comprised cigarette smoking (40 patients, 31.8%) and alcohol drinking (42 patients, 33.3%). Previous specific oral medications included statins (28 patients, 22.2%), anticoagulation drugs (8 patients, 6.4%), and antiplatelet drugs (17 patients, 13.5%). The median time was 12.0 h (range, 0.5–24.0 h; lower-upper quartiles, 7.5–18.8 h) from stroke onset to hospital admission, and the median time was 14.0 h (range, 1.0–29.0 h; lower-upper quartiles, 9.0–20.5 h) from stroke onset to blood acquirement. Mean values of systolic arterial pressure, diastolic arterial pressure, and mean arterial pressure were 162.2 ± 24.3 mmHg, 90.0 ± 17.0 mmHg, and 114.1 ± 17.9 mmHg, respectively. Lobar and infratentorial hemorrhages were found in 26 (20.6%) and 19 patients (15.1%), respectively. Extensions of hematomas into the intraventricular cavity and subarachnoidal space were revealed in 22 (17.5%) and 9 patients (7.1%), respectively. Median values of NIHSS score, GCS score, and ICH score were 9 (lower-upper quartiles, 4–14), 12 (lower-upper quartiles, 9–14), and 1 (lower-upper quartiles, 0–2), respectively. Median hematoma volume was 12 ml (lower-upper quartiles, 7–22 ml). In aggregate, 36 patients (28.6%) experienced END, and 54 patients (42.9%) had an unfavorable outcome at post-stroke 90 days.

### Serum ANXA7 levels and hemorrhagic severity

**Figure 2:**
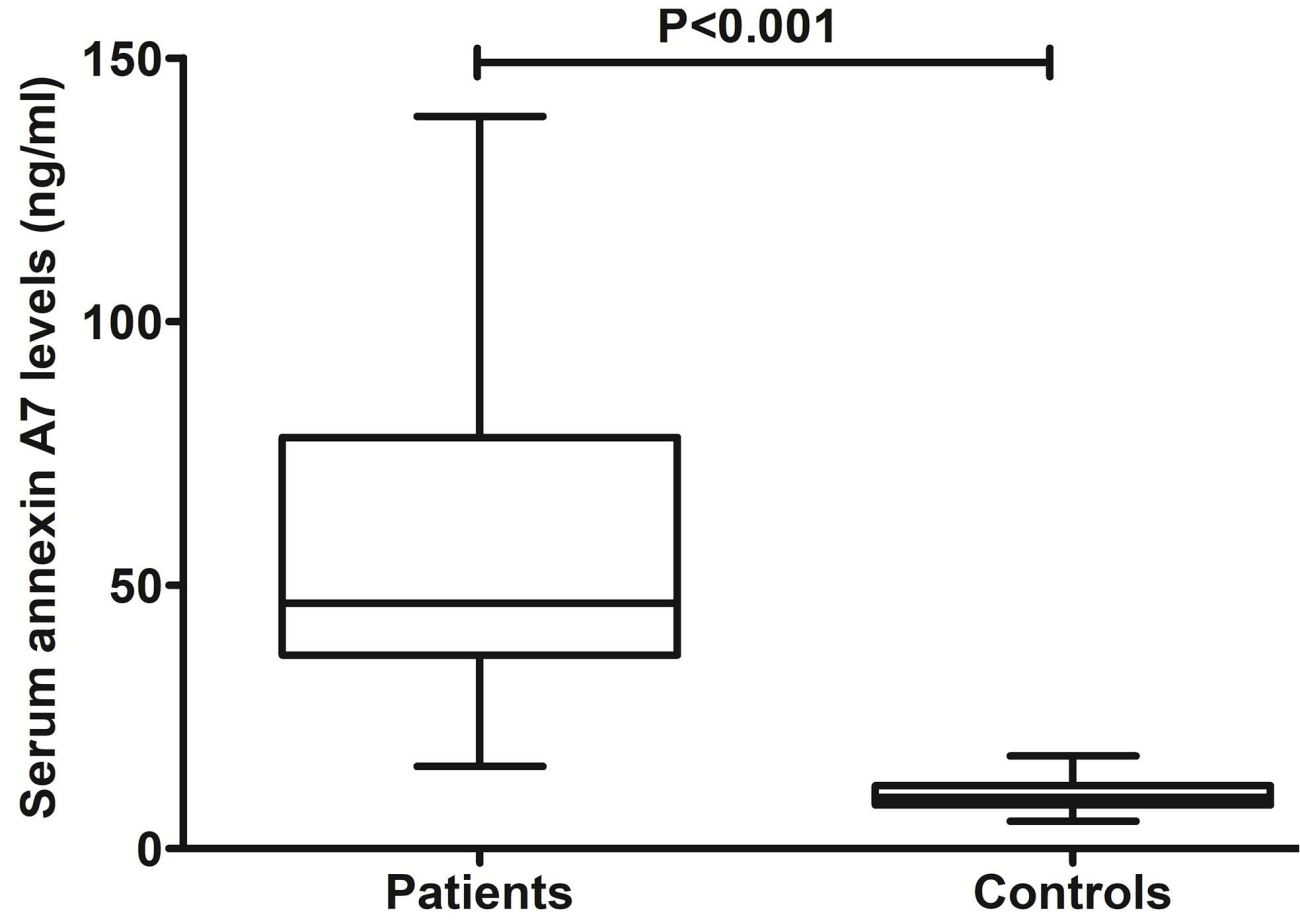
Change of serum annexin A7 levels after spontaneous intracerebral hemorrhage. There was a substantial enhancement in serum annexin A7 levels of patients with intracerebral hemorrhage relative to healthy controls using the Mann–Whitney *U* test (*P* < 0.001).

**Figure 3:**
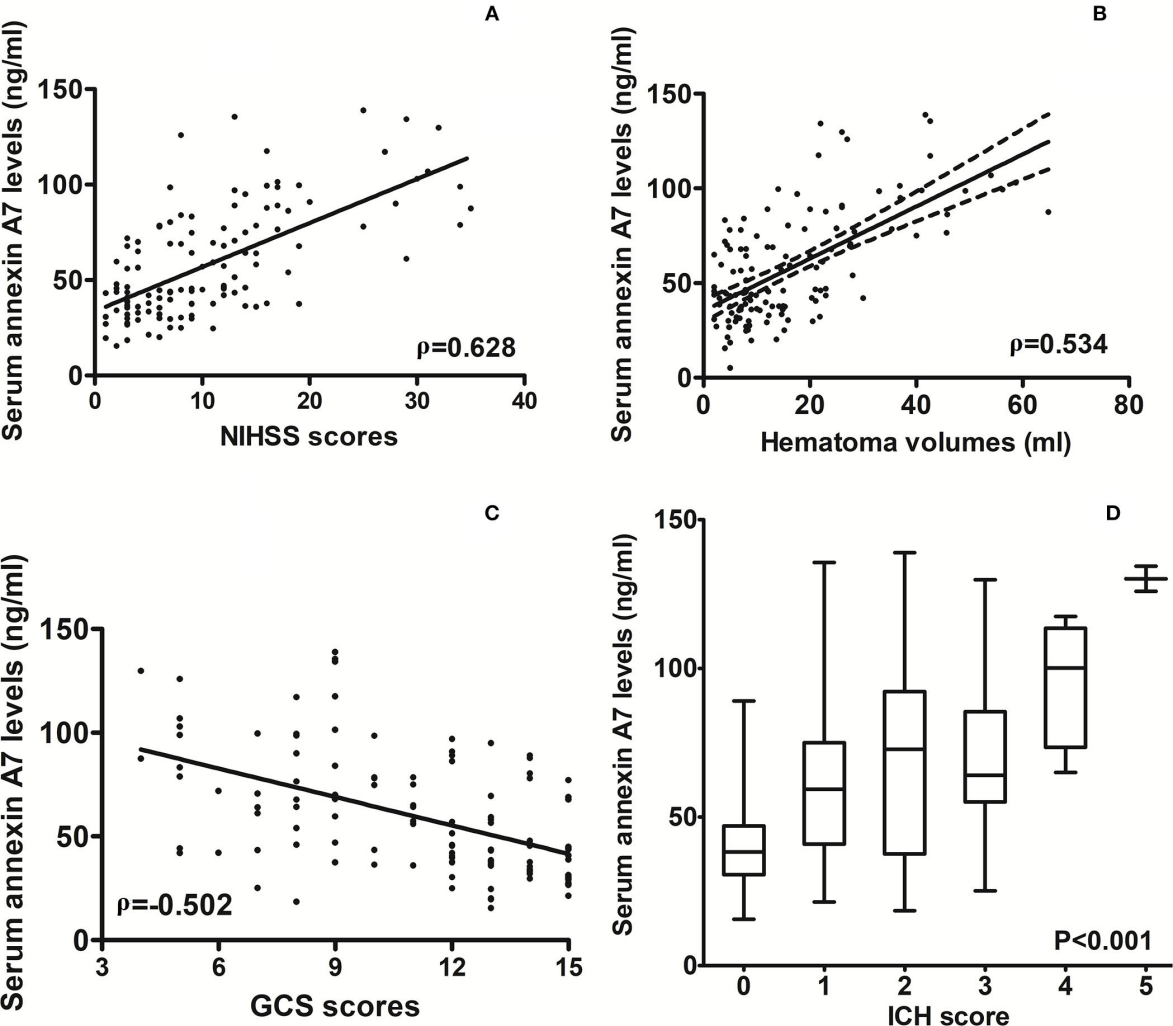
Relationship between serum annexin A7 levels and illness severity after spontaneous intracerebral hemorrhage. **(A)** Correlation of serum annexin A7 levels with National Institutes of Health Stroke Scale scores following acute intracerebral hemorrhage. **(B)** Relationship between serum annexin A7 levels and hematoma volume after acute intracerebral hemorrhage. **(C)** Relation of serum annexin A7 levels to Glasgow Coma Scale scores after acute intracerebral hemorrhage. **(D)** Differences in serum annexin A7 levels across intracerebral hemorrhage scores. Bivariate correlations were analyzed using the Spearman's correlation coefficient, and associations were reported as *r* values. Comparison of data was done among multiple groups using the Kruskal–Wallis *H* test. Graphs show that serum annexin A7 levels were significantly associated with stroke severity (all *P* < 0.001). NIHSS, National Institutes of Health Stroke Scale; GCS, Glasgow Coma Scale; ICH, intracerebral hemorrhage.

As compared to healthy controls, there was a significant elevation of serum ANXA7 levels in ICH patients (Figure 2, *P* < 0.001). In this study, NIHSS scores, GCS scores, hematoma volume, and ICH scores were estimated as the severity indicators of ICH. Serum ANXA7 levels were strongly correlated with NIHSS scores (Figure 3A, *P* < 0.001), hematoma volume (Figure 3B, *P* < 0.001), and GCS scores (Figure 3C, *P* < 0.001); serum ANXA7 levels were significantly raised with increasing ICH scores (Figure 3D, *P* < 0.001).

Table 1 shows that serum ANXA7 levels were closely correlated with diabetes mellitus, extension of hematoma into the intraventricular cavity, extension of hematoma into subarachnoidal space, NIHSS scores, hematoma volume, GCS scores, ICH scores, serum glucose levels, and serum C-reactive protein levels (all *P* < 0.05). When the aforementioned variables, except ICH scores, were forced into the multivariate linear regression model, we found that serum ANXA7 levels were independently correlated with NIHSS scores (beta: 0.821; 95% CI: 0.106–1.514; variance inflation factor: 5.180; *t* = 2.573; *P* = 0.014) and hematoma volume (beta: 0.794; 95% CI: 0.418–1.173; variance inflation factor: 5.281; *t* = 2.781; *P* = 0.007).

**Table 1:** Correlated factors of serum annexin A7 levels after acute intracerebral hemorrhage.

**Variables**	**ρ**	***P*-value**
Age (years)	0.151	0.091
Gender (male/female)	0.134	0.136
Body mass index (kg/m^2^)	0.143	0.109
Hypertension	0.017	0.851
Diabetes mellitus	0.195	0.028
Hyperlipidemia	0.093	0.298
Current smoking	0.063	0.483
Alcohol consumption	0.122	0.174
Previous use of statins	0.081	0.367
Previous use of anticoagulation drugs	−0.167	0.062
Previous use of antiplatelet drugs	0.011	0.901
Time from onset to inclusion (h)	0.108	0.228
Time from onset to blood-collection (h)	0.123	0.172
Systolic arterial pressure (mmHg)	0.163	0.067
Diastolic arterial pressure (mmHg)	0.102	0.254
Mean arterial pressure (mmHg)	0.119	0.183
Lobar hemorrhage	0.115	0.201
Infratentorial hemorrhage	0.159	0.076
Intraventricular hemorrhage	0.427	<0.001
Subarachnoidal hemorrhage	0.274	0.002
NIHSS score	0.628	<0.001
Hematoma volume (ml)	0.534	<0.001
ICH score	0.491	<0.001
Glasgow coma scale score	−0.502	<0.001
Blood leucocyte count (×10^9^/l)	0.152	0.088
Serum glucose levels (mmol/l)	0.419	<0.001
Serum C-reactive protein levels (mg/l)	0.371	<0.001

Statistical analysis was done using the Spearman's correlation coefficient. NIHSS, National Institutes of Health Stroke Scale; ICH, intracerebral hemorrhage.

### Serum ANXA7 levels and END

**Figure 4:**
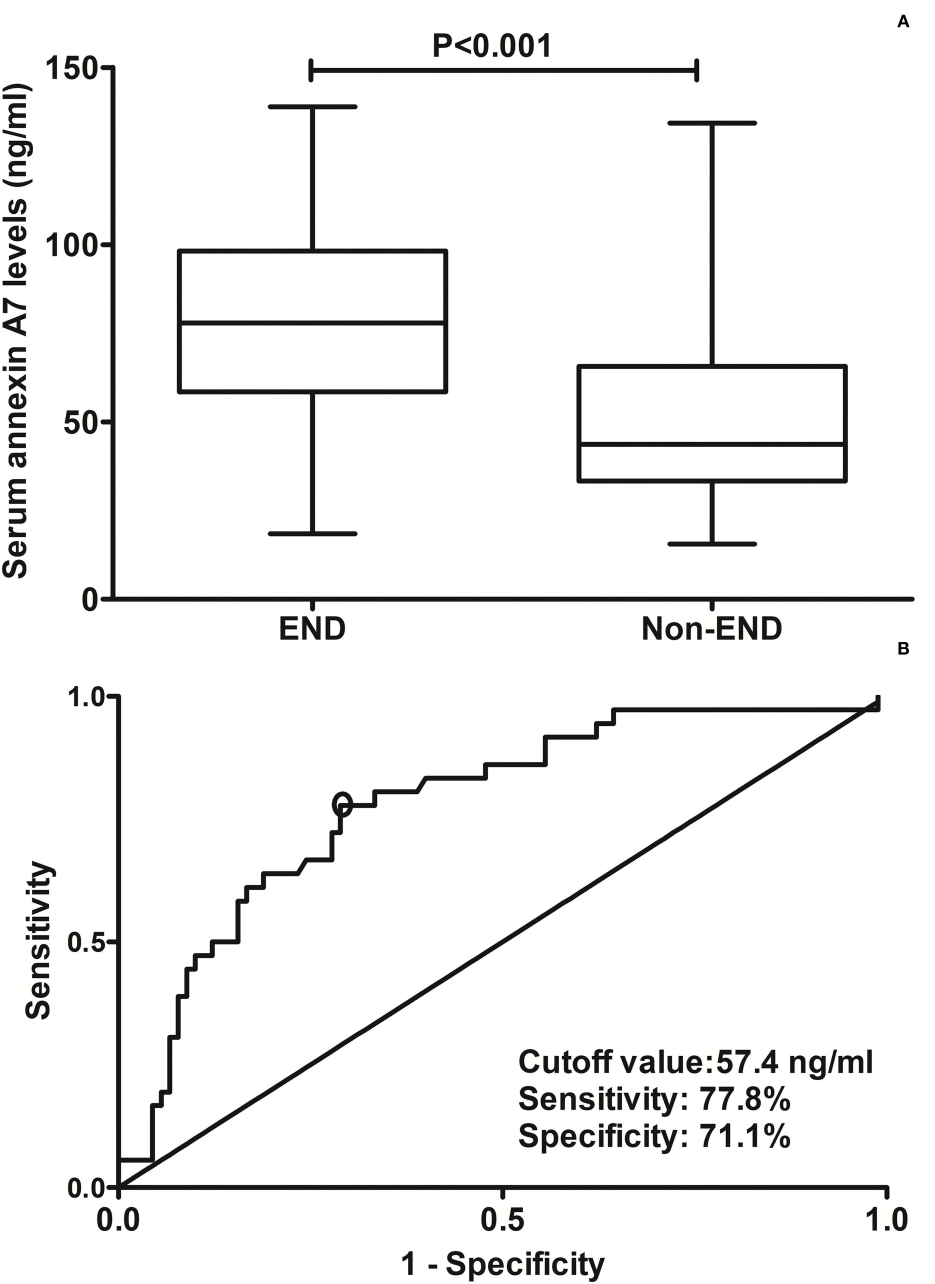
Analysis regarding the predictive value of serum annexin A7 levels for early neurological deterioration after acute intracerebral hemorrhage. **(A)** Comparison of serum annexin A7 levels between patients with development of early neurological deterioration and those not presenting with early neurological deterioration. **(B)** Assessment of the discriminatory ability of serum annexin A7 levels for early neurological deterioration under the receiver operating characteristic curve. Serum annexin A7 levels were dramatically higher in patients suffering from early neurological deterioration than in those who did not using Mann–Whitney *U* test (*P* < 0.001). Serum annexin A7 levels significantly discriminate risk of early neurological deterioration with an area under the curve at 0.781 (95% confidence interval: 0.698–0.849). Using maximum Youden index, serum annexin A7 levels above 57.4 ng/ml differentiated development of END with medium-high sensitivity and specificity. END, early neurological deterioration.

In Figure 4A, serum ANXA7 levels were substantially higher in patients with development of END than in those not suffering from END (*P* < 0.001). Serum ANXA7 levels had significantly efficient discriminatory ability for patients at risk of END with an AUC of 0.781 (95% CI: 0.698–0.849). Using the maximum Youden index (namely, 0.489), 57.4 ng/ml was selected as an optimal cutoff value of serum ANXA7 levels, which differentiated patients who experienced END with medium-high sensitivity and specificity (Figure 4B).

Table 2 shows that, as compared to patients not experiencing END, those at risk of END had significantly raised possibility of serum annexin A7 levels >57.4 ng/ml and extension of hematoma into intraventricular cavity, tended to display substantially increased NIHSS scores, hematoma volume, ICH scores, blood leucocyte count, blood glucose levels, and serum C-reactive protein levels, as well as exhibited markedly decreased GCS scores (all *P* < 0.05). Using multivariate analysis, serum annexin A7 levels >57.4 ng/ml independently predicted END after adjustment for intraventricular hemorrhage, NIHSS scores, hematoma volume, and GCS scores (Table 3).

**Table 2:** Related factors of early neurological deterioration in acute intracerebral hemorrhage.

**Variables**	**Early neurologic deterioration**		**P Value**
	**Presence (*n* = 36)**	**Absence (*n* = 90)**	
Age (years)	62.1 ± 10.4	58.7 ± 10.9	0.107
Gender (male/female)	21/15	49/41	0.691
Body mass index (kg/m^2^)	25.4 ± 1.7	24.8 ± 1.9	0.110
Hypertension	26 (72.2%)	52 (57.8%)	0.131
Diabetes mellitus	8 (22.2%)	12 (13.3%)	0.217
Hyperlipidemia	12 (33.3%)	24 (26.7%)	0.454
Current smoking	8 (22.2%)	32 (35.6%)	0.146
Alcohol consumption	13 (36.1%)	29 (32.2%)	0.676
Previous use of statins	9 (25.0%)	19 (21.1%)	0.635
Previous use of anticoagulation drugs	1 (2.8%)	7 (7.8%)	0.438
Previous use of antiplatelet drugs	8 (22.2%)	9 (10.0%)	0.086
Time from onset to inclusion (h)	12.0 (6.0–17.5)	12.0 (8.0–20.0)	0.422
Time from onset to blood-collection (h)	13.0 (8.0–20.0)	14.0 (9.0–22.0)	0.276
Systolic arterial pressure (mmHg)	165.4 ± 27.6	160.8 ± 22.8	0.337
Diastolic arterial pressure (mmHg)	92.8 ± 23.3	88.9 ± 13.6	0.350
Mean arterial pressure (mmHg)	117.0 ± 23.5	112.9 ± 15.1	0.242
Lobar hemorrhage	5 (13.9%)	21 (23.3%)	0.237
Infratentorial hemorrhage	4 (11.1%)	15 (16.7%)	0.431
Intraventricular hemorrhage	19 (25.0%)	3 (3.3%)	<0.001
Subarachnoidal hemorrhage	3 (8.3%)	6 (6.7%)	0.714
NIHSS score	15 (12–18)	7 (3–12)	<0.001
Hematoma volume (ml)	27.8 (15.4–42.1)	8.7 (6.2–15.0)	<0.001
ICH score	2 (1–3)	0 (0–1)	<0.001
Glasgow coma scale score	8 (7–12)	13 (10–14)	<0.001
Blood leucocyte count (×10^9^/l)	9.2 (6.8–13.0)	7.9 (6.4–9.9)	0.041
Serum glucose levels (mmol/l)	11.1 (9.0–12.3)	8.7 (7.5–12.0)	0.032
Serum C-reactive protein levels (mg/l)	16.1 (14.5–21.1)	13.9 (12.0–17.2)	0.015
Serum annexin A7 levels > 57.4 ng/ml	28 (77.8%)	26 (28.9%)	<0.001

Variables were presented as count (percentage), median (upper-lower quartiles), or mean ± standard deviation as appropriate. Statistical methods included the chi-square test, Fisher's exact test, unpaired Student's t-test, and Mann–Whitney U test. NIHSS, National Institutes of Health Stroke Scale; ICH, intracerebral hemorrhage.

**Table 3:** Relation of serum annexin A7 levels to early neurological deterioration using multivariate analysis.

	**B**	**S.E**.	**Wals**	**Odds ratio (95% CI)**	***P***
Model 1	2.154	0.463	21.591	8.615 (3.474–21.369)	<0.001
Model 2	1.376	0.572	5.785	3.958 (1.290–12.143)	0.016

In model 1, results were reported using univariate binary logistic regression analysis. In model 2, results were shown using multivariate binary logistic regression analysis, with adjustment for intraventricular hemorrhage, National Institutes of Health Stroke Scale scores, hematoma volume, and Glasgow Coma Scale score. 95% CI indicates 95% confidence interval.

### Serum ANXA7 levels and 90-day unfavorable outcome

**Figure 5:**
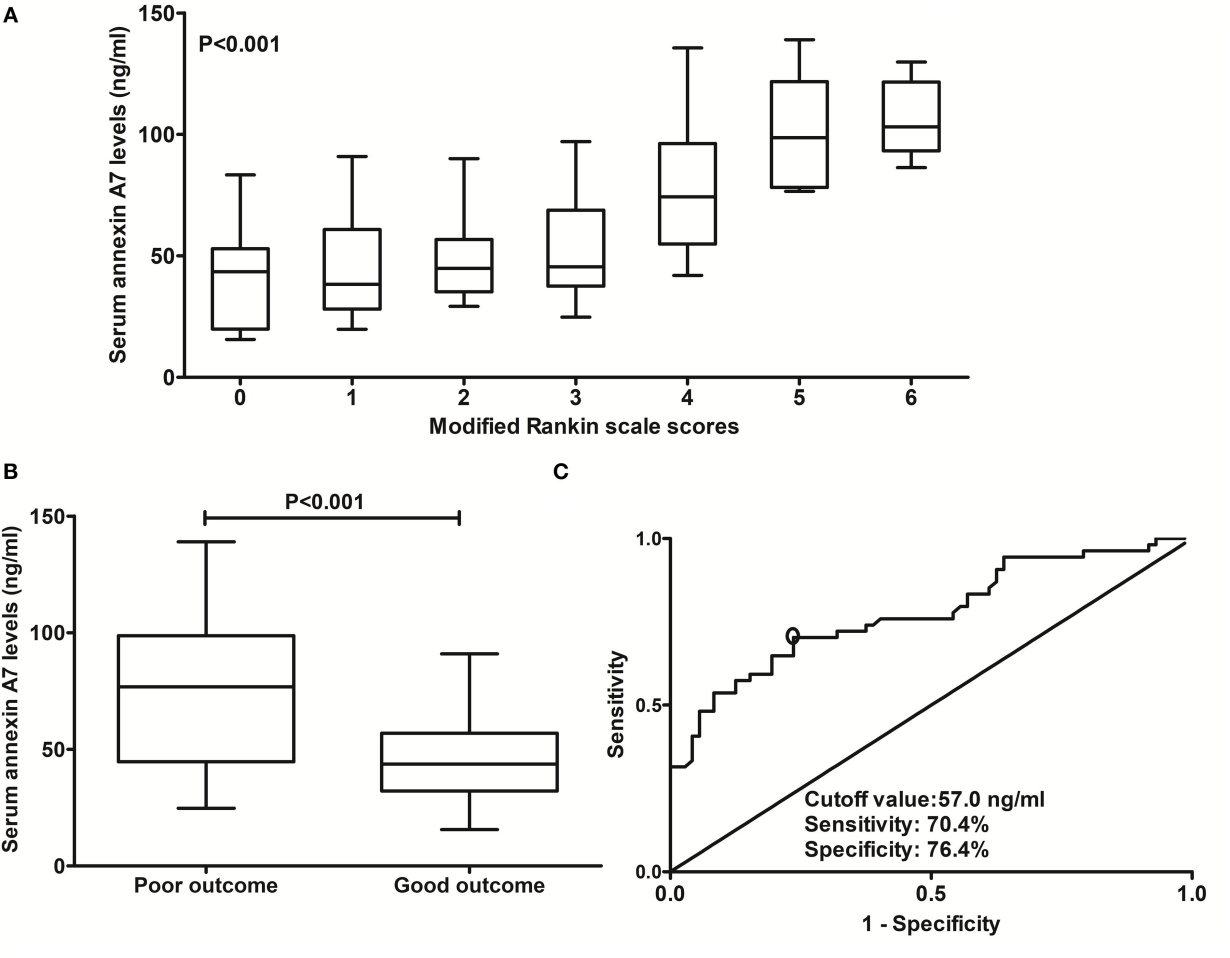
Relationship between serum annexin A7 levels and prognosis at 90 days after intracerebral hemorrhage. **(A)** Relationship between serum annexin A7 levels and modified Rankin Scale score among patients with acute intracerebral hemorrhage. **(B)** Comparison of serum annexin A7 levels between patients with poor outcome and those with a good outcome. **(C)** Analysis with respect to the predictive value of serum annexin A7 levels for 90-day poor outcome after acute intracerebral hemorrhage. Comparison of data was done among multiple groups using the Kruskal–Wallis *H* test. There was a significant elevation of serum annexin A7 levels after hemorrhagic stroke by modified Rankin Scale scores (*P* < 0.001). Serum annexin A7 levels were dramatically higher in patients with poor outcome than in those with good outcome using Mann–Whitney *U* test (*P* < 0.001). Serum annexin A7 levels significantly predicted 90-day poor outcome, with area under the curve at 0.776 (95% confidence interval: 0.693–0.846); and serum annexin A7 levels above 57.0 ng/ml predicted a poor outcome with medium-high sensitivity and specificity under the receiver operating characteristic curve. A poor outcome was referred to as a modified Rankin scale score above 2.

In Figure 5A, serum ANXA7 levels were substantially elevated after ICH, with increasing mRS scores at post-stroke 90 days. In addition, patients with mRS scores of 3–6 had significantly higher serum ANXA7 levels than those with mRS scores of 0–2 (Figure 5B, *P* < 0.001). Moreover, serum ANXA7 levels significantly discriminated against patients who experienced 90-day unfavorable outcome, with AUC at 0.776 (95% CI: 0.693–0.846), and serum ANXA7 levels above 57.0 ng/ml predicted patients with an unfavorable outcome with medium-high sensitivity and specificity (Figure 5C).

Just as listed in Table 4, there were substantial differences in terms of age, NIHSS scores, hematoma volume, ICH scores, GCS scores, serum glucose levels, and serum C-reactive protein levels, as well as percentages of serum ANXA7 levels above 57.0 ng/ml and extension of hematoma into the intraventricular cavity (all *P* < 0.05). Using multivariate analysis, serum ANXA7 levels above 57.0 ng/ml were retained as the independent predictor of post-injury 90-day unfavorable outcome after adjustment for age, intraventricular hemorrhage, NIHSS scores, hematoma volume, and GCS scores (Table 5).

**Table 4:** Pertinent factors of 90-day poor outcome after acute intracerebral hemorrhage.

**Variables**	**90-day poor outcome**		***P*-value**
	**Presence (*n* = 54)**	**Absence (*n* = 72)**	
Age (years)	62.5 ± 11.4	57.5 ± 10.0	0.011
Gender (male/female)	29/25	41/31	0.717
Body mass index (kg/m^2^)	25.3 ± 2.1	24.7 ± 1.6	0.059
Hypertension	34 (63.0%)	44 (61.1%)	0.832
Diabetes mellitus	11 (20.4%)	9 (12.5%)	0.232
Hyperlipidemia	15 (27.8%)	21 (29.2%)	0.864
Current smoking	17 (31.5%)	23 (31.9%)	0.956
Alcohol consumption	19 (35.2%)	23 (31.9%)	0.703
Previous use of statins	13 (24.1%)	15 (20.8%)	0.665
Previous use of anticoagulation drugs	2 (3.7%)	6 (8.3%)	0.465
Previous use of antiplatelet drugs	7 (13.0%)	10 (13.9%)	0.880
Time from onset to inclusion (h)	12.0 (7.0–18.5)	11.5 (8.0–19.5)	0.875
Time from onset to blood-collection (h)	14.0 (8.0–20.0)	13.5 (9.0–21.5)	0.910
Systolic arterial pressure (mmHg)	163.5 ± 24.6	161.2 ± 24.2	0.601
Diastolic arterial pressure (mmHg)	92.8 ± 20.7	88.0 ± 13.3	0.112
Mean arterial pressure (mmHg)	116.4 ± 20.5	112.4 ± 15.6	0.216
Lobar hemorrhage	7 (13.0%)	19 (26.4%)	0.065
Infratentorial hemorrhage	8 (14.8%)	11 (15.3%)	0.943
Intraventricular hemorrhage	17 (31.5%)	5 (6.9%)	<0.001
Subarachnoidal hemorrhage	6 (11.1%)	3 (4.2%)	0.134
NIHSS score	13 (11–17)	6 (3–9)	<0.001
Hematoma volume (ml)	21.1 (14.0–36.9)	7.9 (5.0–12.9)	<0.001
ICH score	2 (1–2)	0 (0–1)	<0.001
Glasgow coma scale score	10 (8–12)	14 (10–15)	<0.001
Blood leucocyte count (×10^9^/l)	8.8 (6.8–11.8)	7.9 (6.3–9.7)	0.068
Serum glucose levels (mmol/l)	10.2 (8.1–13.2)	8.6 (7.4–11.4)	0.029
Serum C-reactive protein levels (mg/l)	15.9 (13.1–21.0)	14.1 (11.8–16.2)	0.017
Serum annexin A7 levels >57.0 ng/ml	38 (70.4%)	17 (23.6%)	<0.001

Variables were presented as count (percentage), median (upper-lower quartiles), or mean ± standard deviation as appropriate. Statistical methods included the chi-square test, Fisher's exact test, unpaired Student's t-test, and Mann–Whitney U test. NIHSS, National Institutes of Health Stroke Scale; ICH, intracerebral hemorrhage.

**Table 5:** Relation of serum annexin A7 levels to 90-day poor outcome using multivariate analysis.

	**B**	**S.E**.	**Wals**	**Odds ratio (95% CI)**	***P***
Model 1	2.039	0.407	25.075	7.684 (3.459–17.069)	<0.001
Model 2	1.013	0.492	4.248	2.755 (1.051–7.220)	0.039

In model 1, results were reported using univariate binary logistic regression analysis. In model 2, results were shown using multivariate binary logistic regression analysis, with adjustment for age, intraventricular hemorrhage, National Institutes of Health Stroke Scale scores, hematoma volume, and Glasgow Coma Scale scores. 95% CI indicates 95% confidence interval.

## Discussion

To the best of our knowledge, this is the first series investigating the relationship between serum ANXA7 levels and severity plus prognosis after acute brain injury as well as subsequently showing the interesting results. The main findings of our study were that (1) there was a marked enhancement in serum ANXA7 levels after ICH, as compared to healthy controls; (2) serum ANXA7 levels were substantially correlated with NIHSS scores, bleeding size, GCS scores, and ICH scores following ICH; (3) serum ANXA7, which was identified as a categorical variable, remained as an independent predictor for END and 90-day unfavorable outcome after ICH; and (4) serum ANXA7 levels efficiently differentiated patients with END or 90-day unfavorable outcome among ICH patients. Overall, serum ANXA7 may be a promising biochemical variable for assessing stroke severity and predicting clinical outcome.

ANXA7 was predominantly located in neurons of normal human or animal cerebral cortices (11–15). Moreover, its expression was greatly upregulated not only in brain tissues of rats with traumatic brain injury (11), ICH (12), subarachnoid hemorrhage (13), or acquired epilepsy (14) but also in those of refractory epilepsy patients (15). Specifically, ANXA7 protein expression was increased significantly in brain tissues of rats with acquired epilepsy at 6, 24, and 48 h after the onset of seizure (14). In neurons of rats with ICH, mRNA levels of ANXA7 were raised substantially from 6 h after ICH, reached the highest point at 24 h, and then declined gradually (12). After traumatic brain injury, expression levels of rat cortical ANXA7 increased during the 6-h period immediately, peaked in 24 h, plateaued at 48 h, decreased gradually thereafter, and was substantially higher than those in sham-operational rats during the 72-h period (11). Also, ANXA7 protein expression was enhanced markedly at 24 h following experimental subarachnoid hemorrhage (13). There was the median time of 14.0 h from stroke onset to blood acquirement in this study. We found a significant elevation of ANXA7 levels in the peripheral blood of humans with ICH. Taken together, ANXA7 expression is presumed to increase significantly in the acute period after acute brain injury. Because blood–brain barrier permeability is obviously enhanced after ICH (21), ANXA7 may be increasingly released from the central nervous system. Collectively, increased ANXA7 levels in the peripheral blood may be at least partially attributed to its release from brain tissues after ICH.

ANXA7 mainly functions in accordance with the Ca^2+^ concentration (22). And, *via* binding to the cell membrane, it can change the permeability of the cell membrane, thereby facilitating the release of neurotransmitters and vesicle transport (23). In rats with traumatic brain injury, the inhibition of ANXA7 using small interfering RNA significantly decreased the expression of A7 in the injured brain tissue, as well as also depressed brain edema, lessened blood–brain barrier permeability, attenuated cell death, and reduced neuronal apoptosis, as compared to the sham rats (11). In rats with subarachnoid hemorrhage, ANXA7 knockdown markedly reduced early brain injury *via* alleviating disruption of blood–brain barrier, ameliorating brain edema, and depressing neuronal apoptosis (13). Glutamate, *via* binding to N-methyl-D-aspartate (NMDA) receptors, induces excitotoxicity (24). ANXA7 knockdown dramatically decreased glutamate release from injured brain tissues in rats with subarachnoid hemorrhage (13). Synaptosome associated protein 25 (SNAP25) and SNAP23 are involved in presynaptic glutamate release and post-synaptic glutamate receptor (NMDA receptor) trafficking, respectively (25, 26). ANXA7 interacted with SNAP25 at presynaptic axon terminals and SNAP23 at post-synaptic axon terminals, subsequently mediating glutamate toxicity after ICH (12). Taken together, ANXA7 may aggravate secondary brain injury at least partially *via* promoting excitatory neurotoxicity; and ANXA7-targeting therapy may be a promising approach to attenuate secondary brain injury.

High expression of ANXA7 was intimately related to the poor neurological status of subarachnoid hemorrhage rats (13). However, it remains unclear whether circulating ANXA7 levels are associated with hemorrhagic severity and clinical outcome after acute brain injury. Generally, the rates of END and unfavorable outcome were ~ 30 and 45%, respectively (7, 8, 27, 28). In our study, 36 patients (28.6%) experienced END, and 54 patients (42.9%) had an unfavorable outcome at post-stroke 90 days. Hence, our data about rates of END and unfavorable outcome after ICH were in line with previous reports (7, 8, 27, 28). In addition, 54 patients who developed a poor prognosis at 90 days included 36 patients with END at 24 h and 24 patients were in overlap between the two groups. Using multivariate analysis, our study found some interesting results that serum ANXA7 levels were independently associated with illness severity, END, and 90-day prognosis after ICH. Moreover, under ROC curve, serum ANXA7 levels possessed efficient prognostic predictive ability for END and 90-day poor prognosis, which was defined as mRS scores of 3–6. Such data may be supportive of the notion that serum ANXA7 may be a potential prognostic biomarker of acute brain injury.

There are several limitations to this study. First, when we investigated the relationship between serum ANXA7 levels and prognosis of ICH, we excluded those patients who underwent a surgical evacuation of hematoma in order to decrease the clinical heterogeneity. Although only a very small portion of patients (namely, 12 patients) were excluded from the final analysis, those excluded patients are likely to have developed END and, therefore, their exclusion may represent a selection bias. Second, we used multivariate analysis to demonstrate the relationship serum ANXA7 levels and severity, END, and prognosis after ICH. Compelling data have shown that END is related to poor prognosis (29, 30). It is significant to study the association of serum ANXA7 levels with a poor prognosis of ICH. Alternatively, univariate analysis has 20 variables. However, infection complications, malnutrition, and ischemic or hemorrhagic relapses may be confounding factors, which were not investigated in this study. Hence, a further study that includes such confounding factors is warranted.

## Conclusion

This is the first series to ascertain whether serum ANXA7 levels are associated with stroke severity and clinical outcome of ICH. Using multivariate analysis, it is revealed that rising serum ANXA7 levels are dramatically related to NIHSS scores and hematoma volume, as well as END and 90-day unfavorable outcome after ICH. Hence, it is possible that serum ANAX7 levels can be used to reflect severity and clinical outcome after ICH.

## Data availability statement

The raw data supporting the conclusions of this article will be made available by the authors, without undue reservation.

## Ethics statement

The studies involving human participants were reviewed and approved by the Human Investigations Committee of the Quzhou Affiliated Hospital of Wenzhou Medical University. The patients/participants provided their written informed consent to participate in this study.

## Author contributions

All authors listed have made a substantial, direct, and intellectual contribution to the work and approved it for publication.

## Conflict of interest

The authors declare that the research was conducted in the absence of any commercial or financial relationships that could be construed as a potential conflict of interest.

## Publisher's note

All claims expressed in this article are solely those of the authors and do not necessarily represent those of their affiliated organizations, or those of the publisher, the editors and the reviewers. Any product that may be evaluated in this article, or claim that may be made by its manufacturer, is not guaranteed or endorsed by the publisher.

## Abbreviations

AUC: area under the curve
CI: confidence interval
CT: computerized tomography
ICH: intracerebral hemorrhage
NIHSS: National Institutes of Health Stroke Scale
OR: odds ratio
ROC: receiver operating characteristic
95% CI: 95% confidence interval
ANXA7: annexin A7.
